# Clutter Effect Investigation on Co-Polarized Chipless RFID Tags and Mitigation Using Cross-Polarized Tags, Analytical Model, Simulation, and Measurement

**DOI:** 10.3390/s23177562

**Published:** 2023-08-31

**Authors:** Jahangir Alam, Maher Khaliel, Feng Zheng, Klaus Solbach, Thomas Kaiser

**Affiliations:** 1 Institute of Digital-Signal Processing, University of Duisburg-Essen, 47057 Duisburg, Germany; 2 Benha Faculty of Engineering, Benha University, Benha 13511, Egypt

**Keywords:** chipless RFID, analytical model, clutter, simulation, measurement, RCS-based cross-polarized tag

## Abstract

Chipless radio frequency identification (RFID) technology is expected to replace barcode technology due to its ability to read in non-line-of-sight (NLOS) situations, long reading range, and low cost. Currently, there is extensive research being conducted on frequency-coded (FC) co-polarized radar cross-section (RCS)-based tags, which are widely used. However, detecting co-polarized chipless RFID tags in cluttered environments is still a challenge, as confirmed by measuring two co-polarized tags in front of a perfect metal reflector (30.5cm×22.5cm). To address this challenge, a realistic mathematical model for a chipless RFID system has been developed that takes into account the characteristics of the reader and the tag, as well as reflections from cluttered objects. This extensive mathematical model developed for linear chipless RFID systems in clutter scenarios holds the potential to greatly assist researchers in their exploration of RCS-based tags. By relying solely on simulations, this model provides a tool to effectively analyze and understand RCS-based tags, ultimately simplifying the process of generating more authentic tag designs. This model has been simulated and verified with measurement results by placing a single flat metal reflector behind two co-polarized one-bit designs: a dipole array tag and a square patch tag. The results showed that the interfering signal completely overlaps the ID of the co-polarized tag, severely limiting its detectability. To solve this issue, the proposed solution involves reading the tag in cross-polarization mode by etching a diagonal slot in the square patch tag. This proposed tag provides high immunity to the environment and can be detected in front of both dielectric and metallic objects.

## 1. Introduction

The Internet of Things (IoT) is a network of interconnected objects that communicate and exchange information through computing and communication devices. This technology has revolutionized the way objects interact with each other by enabling them to communicate seamlessly without human intervention [1]. Radio frequency identification (RFID) is a critical technology that facilitates this communication by providing wireless connectivity, automatic object identification, and secure data storage [2]. RFID systems consist of tags and readers, which work together to enable communication between objects. Tags are small electronic devices that contain unique identification information, which can be wirelessly transmitted to a reader. The reader sends an interrogation signal to the tags, which then respond by backscattering the signal and transmitting their unique identification information. This process enables the reader to identify and locate objects with ease, without requiring any direct human involvement [3].

Conventional RFID tags have traditionally been designed for a long reading range and a high encoding capacity, typically consisting of an integrated circuit (IC) and an antenna [4]. While these tags offer numerous benefits, such as improved inventory tracking and supply chain management, their cost is often prohibitive due to the need for an IC. This cost factor has limited the mass deployment of RFID tags in many industries [5]. However, chipless RFID systems offer a promising solution to this issue. Unlike conventional RFID tags, chipless systems do not require an integrated circuit or battery, which significantly reduces their cost [6,7]. Chipless tags can be encoded in different domains such as time, frequency, or spatial domains, and each domain has its own advantages and disadvantages [8,9,10]. FD coding schemes are preferred by researchers due to their simpler coding techniques and higher coding capacity. They are also less susceptible to noise and interference compared to TD coding schemes. In the frequency domain (FD) or Frequency-coded (FC) RFID system, chipless tags are encoded by the presence or absence of peaks or notches in their electromagnetic (EM) spectrum. These peaks or notches represent resonant frequencies of the tag’s resonators, which can be used to represent binary data [11,12,13].

There are two types of FC RFID tags based on their physical structure and functioning mechanisms: retransmission-based tags and radar cross-section (RCS)-based tags [14]. Retransmission-based tags consist of resonators and two orthogonal antennas. The resonators store the identification bits, and the antennas receive and send the interrogation signal to the reader. When the reader sends an interrogation signal, the tag’s antenna receives the signal, which activates the resonator and sends the stored information back to the reader through the other antenna. Retransmission-based tags have high coding capacity and cross-polarized antenna systems that reduce the clutter effect, which is the interference caused by the surrounding environment. However, the additional antennas increase the tag’s dimensions, causing mismatch losses between the antennas and resonators, which reduces the tag’s performance [14,15]. RCS-based tags, on the other hand, have resonators that reflect or absorb the energy of the electromagnetic (EM) wave and generate a peak/notch in the RCS spectrum for each resonator, representing an information bit [16].

RCS-based tags do not require additional antennas, allowing a higher number of bits to be encoded in a smaller dimension. RCS-based tags can be co-polarized or cross-polarized.

**Figure 1:**
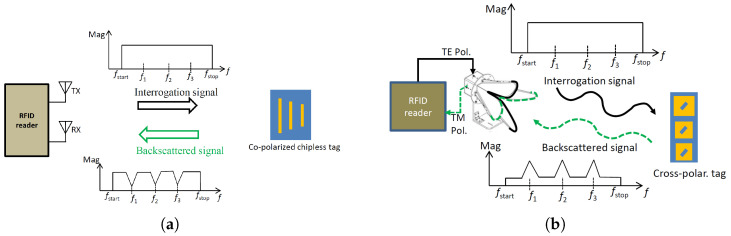
Frequency-coded RCS-based chipless RFID system: (**a**) co-polarization (**b**) cross-polarization.

In a co-polarized RFID system, the reader and tag use signals with the same polarization for transmission and reception, as shown in Figure 1a. This means that the polarization of the incident signal and the backscattered signal are the same. The tag has a certain resonance frequency at which it oscillates, and the backscattered signal contains information encoded in the resonance frequency. When the reader receives the backscattered signal, it shows a notch at the resonance frequency. The reason for this is that the structure mode of the tag, which is related to the physical structure of the tag, dominates the antenna mode in the co-polarization system. The benefits of co-polarized RCS-based tags are that they offer higher RCS levels and more encoding capacity; however, the use of co-polarized RCS-based tags is limited by environmental clutter reflections that interfere with the tag’s reflected signals, resulting in detection errors and reduced reliability [17,18].

On the contrary, the advantage of using cross-polarized RCS-based tags is that they can reduce the clutter effect caused by the surrounding environment. In a cross-polarized RFID system, the reader and tag use signals with different polarizations for transmission and reception, as depicted in Figure 1b. The tag has the ability to alter the polarization of the incident signal, so it backscatters both co-polarization and cross-polarization signals. However, the receiving antenna of the reader is designed to receive only the cross-polarization signal. When the reader receives the backscattered signal, it shows peaks at the resonance frequency. This is because the antenna mode signal dominates the structure mode signal in the cross-polarization system. The reader receives only the cross-polarization signal, which is stronger than the co-polarization signal.

## 2. Related Work

The published literature suggests several techniques for reducing interference in radio frequency identification (RFID) systems. One commonly used technique is empty room calibration, which removes clutter components and self-interference between reader antennas [19]. However, this approach assumes prior knowledge of the channel, which may not be available in practice. Moreover, mobile objects can unexpectedly appear in the channel and produce backscattered signals that cannot be removed by empty room calibration.

Another approach involves filtering out the tag’s ringing response, which contains its unique identification (ID) [20,21]. This method requires pre-knowledge of the time of the scattered signal from the tag compared to the scattered signal from the clutter objects because the distance between the tag and the clutter objects is unknown, and the reflected signals from the objects and tag can overlap, making detection impossible with currently available algorithms. The authors noted in [21] that by analyzing and modeling the frequency and time-domain response of the tag, taking advantage of the time delay between the structural mode signal and the antenna mode signal, a co-polarized RCS-based tag can be detected. Additionally, this method can detect the tag even when it is situated very close to reflectors that are just a few centimeters apart under constrained conditions. However, at specific distances between the reflector and the tag, the structural mode components of the reflectors may unfortunately appear in the antenna mode window of the tag, making detection unreliable. It is important to note that the proposed mathematical model does not consider polarization and angular effects.

To minimize background interference, the researchers in [22] proposed using a cross-polarized tag instead of a co-polarized tag. They suggested two cross-polarized tag designs to eliminate interference effects. The first design, which utilized a double L-shaped resonator, produced resonance harmonics and had a low radar cross-section (RCS). To address these issues, the authors proposed a short dipole array attached to metal coffee cans and water bottles. This design allowed the tag ID to be detected with an incident field tilted by 45°, but this is not always practical in real-world scenarios. Moreover, in this study the impact of interfering signals on the responses of the co-polarized and cross-polarized tags was not explored.

In another study [23], the researchers presented two designs with L-shaped slots that could be printed as a single-layer structure. However, these tags generated unwanted harmonic resonances that restricted the coding capacity.

In order to effectively address all of the issues related to co-polarized tags, it is necessary to develop a model that can investigate their behavior and predict their response. This requires a comprehensive study of the behavior of frequency-coded tags.

To this end, this article presents a detailed study on the behavior of frequency-coded chipless tags using a mathematical model that takes into account various factors such as the angle of arrival, angle of departure, gain, polarization, and clutter effects. By considering these factors, the model can accurately predict the response of the tags and help to identify all potential issues that may arise.

In addition to the comprehensive study, the article proposes a novel cross-polarization tag that utilizes both TE and TM polarizations for interrogation and backscattered signals, respectively. This tag’s cross-polarization response requires a high degree of isolation between the transmit and receive signals in the cross-polarization plane.

The key objectives and contributions of this research can be outlined as follows:

1. The primary objective is to thoroughly investigate the capability of co-polarizing tags in identifying objects within cluttered environments, utilizing both simulations and measurements. The significance of this objective is underscored by the fact that a majority of research articles have predominantly concentrated on co-polarized-based tags.
2. The second objective is to develop a realistic system model for RCS-based tags in cluttered scenarios. This model aims to simulate and characterize the behavior and performance of RCS-based tags within cluttered environments. Key factors to be incorporated in the model include polarization, angle-dependent gain, angle of departure, angle of arrival, RCS model, and clutter reflections.
3. The third goal is to propose and develop a new cross-polarizing tag, demonstrating its immunity to clutter reflections through simulation using an analytical model and actual measurements.

The paper is structured as follows: Section 2 discusses various tag designs and their corresponding analytical models. Section 3 presents the mathematical framework of the FC chipless RFID system based on RCS, taking into account clutter components. Section 4 reports on the simulation and measurement results of co-polarized and cross-polarized tags, both in the presence and absence of clutter. In Section 5, the reliability of detecting co-polarized and cross-polarized tags under different signal distortion factors is evaluated. Lastly, Section 6 provides the conclusion.

## 3. Co- and Cross-Polarized Tag Designs

In this section, our design efforts are focused on creating two co-polarized tags: the shorted dipole array and the square patch tag. Additionally, we modify the square patch tag to operate in a cross-polarized manner. The dipole array tag operates at 4.4GHz, while the patch-based tag operates at 4.2GHz. This configuration is chosen to examine and address the challenges posed by detecting even a single-bit tag at different frequency positions within a cluttered environment. Regarding the cross-polarized tag, we also opted for a single-bit tag configuration to ascertain its capability for detection in cluttered environments. These tags have the advantage of not requiring any additional antennas for reception and reradiation, unlike re-transmission-based tags. Additionally, they can be designed on grounded or non-grounded substrates [14].

### 3.1. Co-Polarized Tag Design

**Figure 2:**
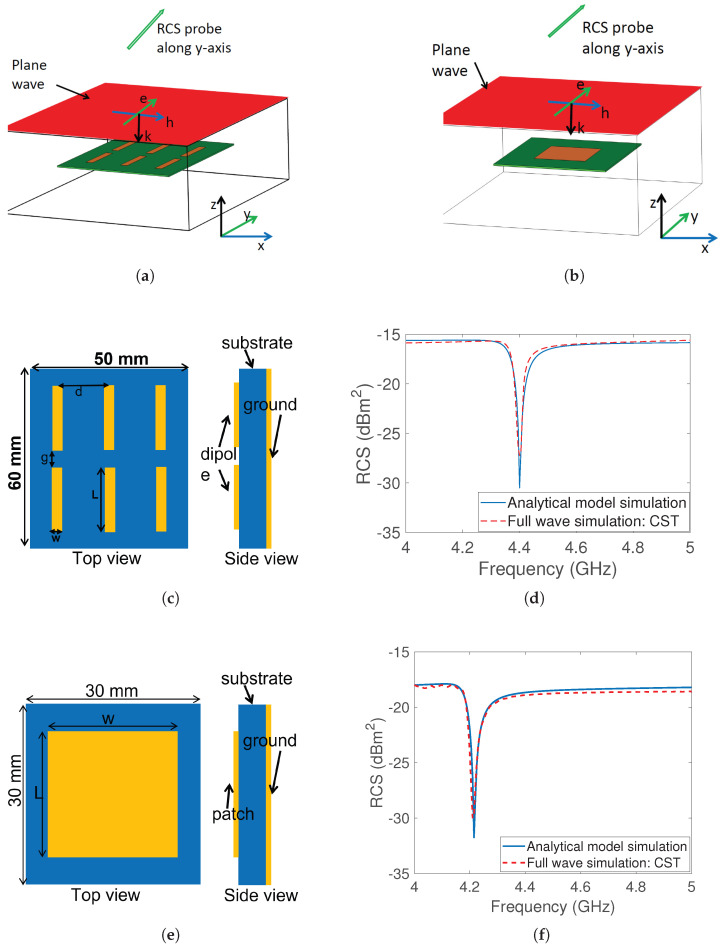
Grounded co-polarized tags: (**a**) CST setup dipole array (plane wave and RCS probe along y-axis) (**b**) CST setup square patch (plane wave and RCS probe along y-axis) (**c**) top and side view of dipole array: L = 17.85mm, w = 2mm, g = 2.5mm and d = 15.5mm (**d**) RCS response of the dipole array using analytical model and CST full wave simulation (**e**) top and side view of square patch: L = 18.25mm, w = 18.25mm (**f**) RCS response of patch tag using analytical model and CST full wave simulation.

The simulation models of grounded co-polarization dipole and square tags are shown in Figure 2a,b. The radar cross-section of the tag was calculated using the electromagnetic simulator, CST Studio Suite. The simulation considered an incident plane wave with an electric field oscillating along the y-axis, as depicted with red plane. To detect the reflected wave, an RCS probe was placed along the y-axis since the tag is co-polarized and both the electric field and the RCS probe are aligned along the y-axis, meaning the orientation of the induced surface current remains unchanged during reflection towards the receiver.

The resonant frequency of the dipole or patch is represented by $f_{r}$ and is calculated based on the speed of light ($c_{0}$), the effective length of the dipole or patch ($L_{\mathrm{eff}}$), and the effective permittivity of the material ($\epsilon_{r,\mathrm{eff}}$). The resonance frequency of both the dipole and patch tags can be calculated using Equation (1) [24]:

(1) $$f_{r}=\frac{c_{0}}{2L_{\mathrm{eff}}\sqrt{\epsilon_{r,\mathrm{eff}}}}$$

The effective length, $L_{\mathrm{eff}}$, is calculated as L+2ΔL, where *L* is the physical length and ΔL represents the extended length caused by the fringing effect. Equations (2) and (3) are used to determine both the effective permittivity and the extended length, respectively [24]:

(2) $$\epsilon_{r,\mathrm{eff}}=\frac{\epsilon_{r}+1}{2}+\frac{\epsilon_{r}-1}{2}{\left[1+12\frac{h}{w}\right]}^{0.5}$$

(3) $$\Delta L=0.0412h\frac{\left(\epsilon_{r,\mathrm{eff}}+0.3\right)\left(\frac{w}{h}+0.264\right)}{\left(\epsilon_{r,\mathrm{eff}}-0.3\right)\left(\frac{w}{h}+0.8\right)}$$

where $\epsilon_{r,\mathrm{eff}}$ is the effective permittivity of the material, *h* is the substrate height, and *w* is the metal strip width.

The substrate material used is RO4003C, which has a permittivity of 3.55 and a loss tangent of 0.0027. Two different frequencies are targeted: 4.4GHz for the dipole array and 4.2GHz for the square patch tag. At the resonant frequency of the resonator, energy absorption results in a notch that serves as a bit of information. The operating frequencies are in the 4–5 GHz range, which is part of the ultra-wideband spectrum and allows for better information encoding. Figure 2c,d depict the layout of both co-polarized tags, while Figure 2e,f display the corresponding RCS outcomes from both EM-simulation and an analytical RCS model.

### 3.2. Cross-Polarized Tag Design

**Figure 3:**
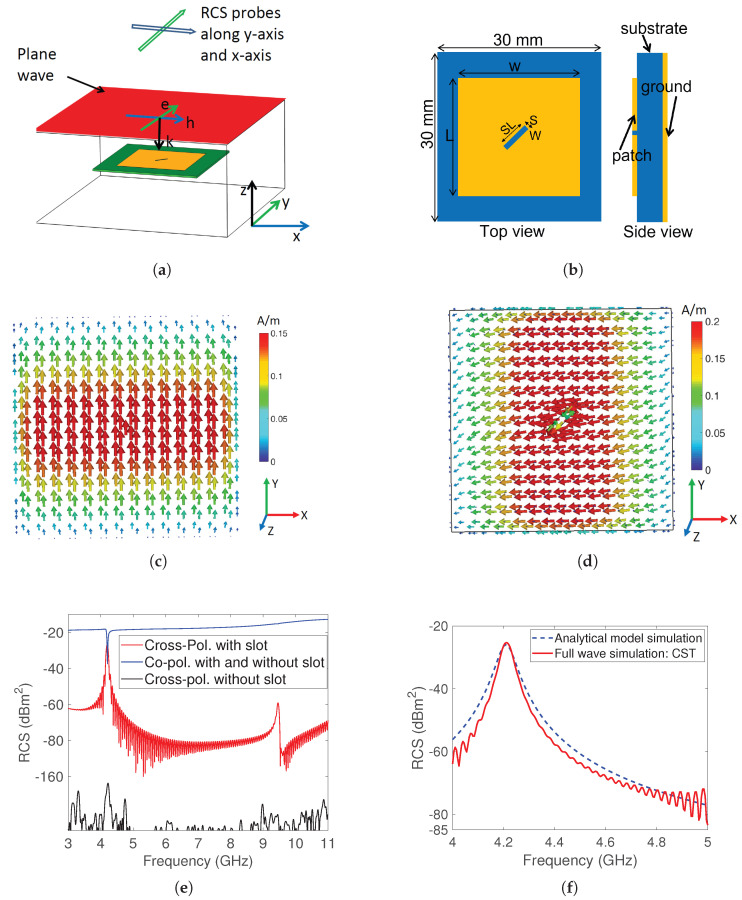
Grounded cross-polarized tag: (**a**) simulation model wherein e, h, and k vectors respectively signify the orientations of the electric field, magnetic field, and wave propagation direction (**b**) top and side view: L = 18.25mm, SL = 4mm and SW = 0.6mm (**c**) surface current distribution for co-polarization (**d**) surface current distribution for cross-polarization (**e**) cross-polarization component enhancement by the slot (**f**) RCS response using CST and analytical model.

This section introduces a new cross-polarizing tag with the aim of enhancing detection accuracy in the presence of environmental reflections. The concept behind reducing clutter is to modify the polarization of the incoming signal using the tag, so that environmental factors cannot affect it, thereby mitigating their impact. The simulation model of the modified design of the square patch tag is shown in Figure 3a.

The asymmetrical design enables us to change the polarization through the implementation of a diagonal slot in the center of the previous patch design. This slotting technique generates cross-polarization and suppresses resonance harmonics. The diagonal slot hinders the current that is induced by incidence, altering its polarization and creating an orthogonal current that boosts cross-polarized radiation. Figure 3c,d show the distribution of the vectorial surface current for co-polarization and cross-polarization, respectively. The RCS response for both co-polarization and cross-polarization is illustrated in Figure 3e. The cross-polarized response without the slot is shown with a black curve, while the response with the slot is depicted with a red curve, demonstrating an improvement in the cross-polarization level of 130 dB. The harmonic effect was also checked and the RCS response was plotted from 3GHz to 11GHz, revealing no harmonic generation in the cross-polarized response, unlike other designs found in the literature [23].

Furthermore, the RCS was modelled analytically and compared with the full-wave simulation results, with close agreement being achieved between the simulation results, as demonstrated in Figure 3f. The results demonstrate that the engineered tag has a peak in the cross-polarization plane at the resonance frequency, along with a notch in the co-polarization plane. The presence or absence of this peak in the cross-polarization plane, dominated by the antenna mode rather than the structural mode, is considered to encode the information bit.

### 3.3. The Analytical Model for the RCS-Based Tags

The definition of RCS is the hypothetical area required to intercept the received power density if the total intercepted power were re-radiated isotropically. If the tag is illuminated by a linearly polarized instantaneous E-field wave ($E_{i}$) placed at distance *d* away, a reflected wave ($E_{r}$) is observed at the receiver. The relationship between the RCS response, incident, and reflected waves is given by [25]:

(4) $$\sigma_{\mathrm{RCS}}\left(f,\psi \right)=\lim_{d\to \infty }4\pi d^{2}\frac{{\left|E_{r}\left(f,\psi \right)\right|}^{2}}{{\left|E_{i}\left(f,\psi \right)\right|}^{2}}$$

where $\sigma_{\mathrm{RCS}}\left(f,\psi \right)$ is the radar cross-section of the tag measured in ${\mathrm{dBm}}^{2}$, *f* is the frequency, ψ represents the transpose vector of excitation and observation angles in degree and is equal to ${\left[\theta_{i},\phi_{i},\theta_{r},\phi_{r}\right]}^{T}$, *d* is the LOS distance between the reader and tag, and $\theta_{i}$ and $\phi_{i}$ are the elevation and azimuth incident angles, respectively; $\theta_{r}$ and $\phi_{r}$ denote the elevation and azimuth reflected angles, respectively.

All the co-polarized RCS-based tags presented in this paper are grounded structures and have two RCS components: structural mode and antenna mode. The metallic ground is mostly responsible for the structural mode which can be modelled by considering a metal-plate and the antenna mode is modelled by a band-reject filter. The total RCS of a grounded co-polarization based chipless tag can be modelled as in (5) [26,27]:

(5) $$\sigma_{\mathrm{RCS},\mathrm{Co}\text{-}P.}\left(f,\psi \right)=\frac{4\pi L_{g}^{2}W_{g}^{2}f^{2}}{c_{0}^{2}}+\frac{A\left(f,\psi \right)f_{r}^{2}}{\left(f_{r}^{2}-f^{2}\right)+jB_{n}f}$$

where $\sigma_{\mathrm{RCS},\mathrm{Co}\text{-}P.}\left(f,\psi \right)$ is the RCS of co-polarized tag, $L_{g}$ and $W_{g}$ are the length and width of the tag’s ground, $A\left(f,\psi \right)=\frac{G(f,\psi )}{Q_{T}}$, G(f,ψ) and $Q_{T}$ are the gain and quality factor at resonance frequency, and $B_{n}$ is the notch bandwidth. However, the structural mode of cross-polarized tags offers a very low cross-polarization component due to its inability to alter the polarization by the structure. Therefore, the cross-polarized tag RCS has only antenna mode and it is modelled by cascading two second-order band-pass filters, as in (6) [26,27]:

(6) $$\sigma_{\mathrm{RCS},\mathrm{Cross}\text{-}P.}\left(f,\psi \right)=A\left(f,\psi \right){\left[\frac{B_{p}f}{\left(f_{r}^{2}-f^{2}\right)jB_{n}f}\right]}^{2}$$

where $\sigma_{\mathrm{RCS},\mathrm{Cross}\text{-}P.}\left(f,\psi \right)$ is the cross-polarized RCS of the tag, $f_{r}$ is the resonance frequency, $B_{p}$ is the peak bandwidth of the tag depending on the quality factor. The peak bandwidth is a function of quality factor, $B_{p}=f_{r}/Q_{T}$, where $Q_{T}$ is the total quality factor of the tag. The quality factor of a grounded printed circuit structure is further calculated by (7) [24]:

(7) $$\frac{1}{Q_{T}}=\frac{1}{Q_{c}}+\frac{1}{Q_{d}}+\frac{1}{Q_{\mathrm{rad}}}$$

where $Q_{c}=h\sqrt{\frac{\pi f\mu }{\rho }}$ represents conductor loss or quality factor, $Q_{d}=\frac{1}{tan\delta }$ represents dielectric loss, $Q_{\mathrm{rad}}=\frac{c_{0}\sqrt{\epsilon_{r,\mathrm{eff}}}}{4hf_{r}}-\frac{\epsilon_{r,\mathrm{eff}}\Delta L}{h}$ represents radiation-loss, *h* is the substrate height, μ is the magnetic permeability, ρ is the resistivity of the conductor, tanδ is the material loss factor, $\epsilon_{r,\mathrm{eff}}$ is the effective permittivity, and ΔL is the extended length by fringing effect.

## 4. RCS-Based FC Chipless RFID System Mathematical Framework

This section presents a comprehensive mathematical framework for the FC chipless RCS-based RFID system, taking into account various practical aspects such as frequency and polarization dependence, angular antenna gain, tag orientation, and clutter reflections. The majority of previous research in the chipless RFID field has primarily concentrated on simulating and measuring tags in scenarios where only a line-of-sight (LOS) path is considered, meaning that no reflector is present in the tag’s environment [1,2,3,4]. In a cluttered environment, the signal transmitted from the chipless RFID reader follows not only the path of reflecting off the tag but also the path of bouncing off various environmental objects, resulting in different propagation paths.

**Figure 4:**
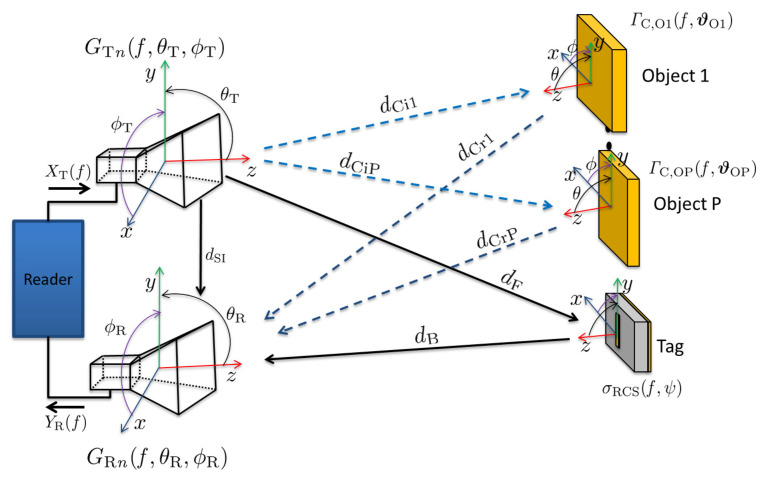
RCS-Based FC Chipless RFID System: Clutter reflections scenario.

The chipless RFID reader system can be configured as either monostatic [1] or bistatic [4]. In this framework, the bistatic configuration is considered as it introduces more complexity and realism to the environment. However, it can be easily adapted to the monostatic configuration by incorporating the same clutter and multipath propagation paths in both the forward and backward channels. Figure 4 provides an illustration of the clutter reflection scenarios in the bistatic configuration.

### 4.1. Reflector Model

The reflection from a clutter object is characterized by the reflection coefficient, as described in Equation (8). This equation assumes that the object size is considerably larger than the wavelength of the incident EM-wave.

(8) $$\Gamma \left(f,\vartheta \right)=\frac{E_{r}\left(f,\vartheta \right)}{E_{i}\left(f,\vartheta \right)}$$

where $E_{r}\left(f,\vartheta \right)$ and $E_{i}\left(f,\vartheta \right)$ denote the reflected and incident electric field intensity, respectively, and ϑ denotes the transpose vector of incident and reflected angles. The reflection coefficient depends on the incidence angle, object materials, frequency, and polarization of the incident wave and can be defined for the TE and TM incidence polarization by (9), (10), respectively [28,29]:

(9) $$\Gamma^{⊥}\left(f,\theta_{i}\right)=\frac{\epsilon_{c}\left(f\right)\sin\theta_{i}-\sqrt{[\epsilon_{c}\left(f\right)-\cos^{2}\theta_{i}}]}{\epsilon_{c}\left(f\right)\sin\theta_{i}+\sqrt{[\epsilon_{c}\left(f\right)-\cos^{2}\theta_{i}}]}$$

(10) $$\Gamma^{\|}\left(f,\theta_{i}\right)=\frac{\sin\theta_{i}-\sqrt{[\epsilon_{c}\left(f\right)-\cos^{2}\theta_{i}}]}{\sin\theta_{i}+\sqrt{[\epsilon_{c}\left(f\right)-\cos^{2}\theta_{i}}]}$$

where ⊥ and ‖ represent TE and TM polarization, respectively, $\epsilon_{c}$ is the complex permittivity of the reflector material, and $\theta_{i}$ is the incident angle with respect to the normal of the reflector. The complex permittivity can be calculated by the following equation [30]:

(11) $$\epsilon_{c}\left(f\right)=\epsilon_{0}\epsilon_{r}\left(f\right)-\frac{j}{2\pi \rho f}$$

where $\epsilon_{r}$ is the relative permittivity of the reflecting surface material, ρ is the resistivity of the reflecting material, and $\epsilon_{0}$ is the permittivity of the vacuum.

When the size of a clutter object is not significantly larger than the wavelength of the incident EM-wave, its reflection is characterized by the radar cross-section (RCS). The RCS of a flat-metal reflector can be represented using the following equation [26,31]:

(12) $$\sigma_{\mathrm{RCS},\mathrm{Ref}.}\left(f,\psi \right)=4\pi {\left(\frac{LWf}{c_{0}}\right)}^{2}\cos^{2}\left(\theta_{r}\right){\left[\frac{\sin\left[\frac{\beta W}{2}\left(\sin\theta_{r}\mp \sin\theta_{i}\right)\right]}{\frac{\beta W}{2}\left(sin\theta_{r}\mp \sin\theta_{i}\right)}\right]}^{2}$$

where “-” for $\phi_{r}=\pi /2$, $0\leq \theta_{r}\leq \pi /2$ and “+” for $\phi_{r}=3\pi /2$, $0\leq \theta_{r}\leq \pi /2$. β is the phase constant, *L* is the length of the reflector, *W* is the width of the reflector, and *f* is the frequency of the incident wave, $\sigma_{\mathrm{RCS},\mathrm{Ref}.}\left(f,\psi \right)$ is the RCS of the reflector, and it is assumed that ψ=ϑ.

The Equation (13) establishes a mathematical relationship between the radar cross-section and the reflection coefficient, as follows [32]:

(13) $$\Gamma_{On}^{⊥,\|}\left(f,\vartheta_{On}\right)=\frac{d_{in}+d_{rn}}{d_{in}d_{rn}}\sqrt{\frac{\sigma_{\mathrm{RCS},\mathrm{Ref}.}\left(f,\psi \right)}{4\pi }}$$

where $d_{i}$ denotes the distance between the transmitter antenna and the reflector, and $d_{r}$ denotes the distance between the reflector and receiver antenna.

### 4.2. The Channel Model

The transmitter emits a signal $X_{T}\left(f\right)$ that is modulated by step frequency continuous wave (SFCW). The transmitting antenna radiates the signal to the tag and to the surrounding objects in the environment. So, we have to consider not only the direct path between the reader to tag, but also the reflection from these objects in the environment.

#### 4.2.1. LOS Model

For the scenario illustrated in Figure 4, there are three LOS signal components: transmitter to tag, tag to receiver, and transmitter to receiver self interference. The LOS forward transfer function for RCS-based tag, i.e., from the transmitter to tag, is modelled by (14):

(14) $$H_{F,\mathrm{LOS}}^{⊥,\|}=\sqrt{G_{T}^{⊥,\|}\left(f,\theta_{T},\phi_{T}\right)\frac{1}{4\pi d_{F}^{2}}}\sqrt{\sigma_{\mathrm{RCS}}^{⊥,\|}\left(f,\psi \right)}\;\;e^{-j2\pi f\frac{d_{F}}{c_{0}}}$$

where $d_{F}$ is the LOS distance between the transmitter antenna and the tag, $G_{T}^{⊥,\|}\left(f,\theta_{T},\phi_{T}\right)$ is the power gain of transmitter antenna, $\sigma_{\mathrm{RCS}}^{⊥,\|}\left(f,\psi \right)$ is the tag RCS which is frequency and angle dependent. The transfer function for the backward LOS channel, i.e., from the tag to receiver is defined by (15):

(15) $$H_{B,\mathrm{LOS}}^{⊥,\|}=\sqrt{\frac{1}{4\pi d_{B}^{2}}A_{\mathrm{eff},R}^{⊥,\|}\left(f,\theta_{R},\phi_{R}\right)}\;\;e^{-j2\pi f\frac{d_{B}}{c_{0}}}$$

where $d_{B}$ is the LOS distance from the tag to the receiver antenna and $A_{\mathrm{eff},R}^{⊥,\|}\left(f,\theta_{R},\phi_{R}\right)$ is the effective area of the receiver antenna which can be expressed by (16):

(16) $$A_{\mathrm{eff},R}^{⊥,\|}\left(f,\theta_{R},\phi_{R}\right)=\frac{\lambda^{2}}{4\pi }G_{R}^{⊥,\|}\left(f,\theta_{R},\phi_{R}\right)$$

where $G_{R}^{⊥,\|}\left(f,\theta_{R},\phi_{R}\right)$ is the receiver antenna gain, and λ is the wavelength of the operating frequency.

The reader LOS self interference can be expressed by (17):

(17) $$H_{\mathrm{SI}}^{⊥,\|}=\sqrt{G_{T}^{⊥,\|}\left(f,\theta_{T},\phi_{T}\right)\frac{1}{4\pi d_{\mathrm{SI}}^{2}}}\sqrt{\frac{\lambda^{2}}{4\pi }G_{R}^{⊥,\|}\left(f,\theta_{R},\phi_{R}\right)}\;\;e^{-j2\pi f\frac{d_{\mathrm{SI}}}{c_{0}}}$$

where $d_{\mathrm{SI}}$ is the distance between the transmitter and receiver antennas.

#### 4.2.2. Clutter Model

**Figure 5:**
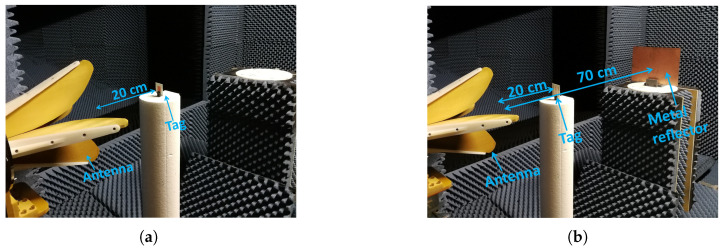
Measurement setup inside the anechoic chamber: (**a**) Tag alone: no presence of the metal reflector (**b**) Tag in presence of the metal reflector at various locations.

The total transfer function from transmitter terminal to receiver terminal for clutter scenario is illustrated in Figure 5 and expressed by (18):

(18) $$H_{\mathrm{Clutter}}^{⊥,\|}=\left[H_{F,\mathrm{LOS}}^{⊥,\|}H_{B,\mathrm{LOS}}^{⊥,\|}\right]+H_{\mathrm{SI}}^{⊥,\|}+H_{C}^{⊥,\|}$$

where $H_{\mathrm{Clutter}}^{⊥,\|}$ is the total transfer function of the channel under the clutter scenario, $H_{F,\mathrm{LOS}}^{⊥,\|}$ is the LOS forward transfer function which includes the RCS of the tag, $H_{B,\mathrm{LOS}}^{⊥,\|}$ is the LOS backward transfer function that includes the receiver’s property, $H_{\mathrm{SI}}^{⊥,\|}$ is the transfer function for the self interference, $H_{C}^{⊥,\|}$ represents the transfer function of all clutter objects, ⊥ and ‖ represent TE and TM polarization, respectively.

Considering that the clutter objects are electrically much larger than the wavelength of the incidence wave, the clutter transfer function for P number of objects can thus be defined by (19):

(19) $$H_{C}^{⊥,\|}=\sum_{n=1}^{P}\sqrt{G_{Tn}^{⊥,\|}\left(f,\theta_{T},\phi_{T}\right)}\left(\frac{\lambda }{4\pi }\right)\frac{\Gamma_{C,On}^{⊥,\|}\left(f,\vartheta_{On}\right)}{d_{\mathrm{Ci}n}+d_{\mathrm{Cr}n}}\sqrt{G_{R\;n}^{⊥,\|}\left(f,\theta_{R},\phi_{R}\right)}\;\;e^{-j2\pi f\frac{\left(d_{\mathrm{Ci}n}+d_{\mathrm{Cr}n}\right)}{c_{0}}}$$

where $G_{Tn}^{⊥,\|}\left(f,\theta_{T},\phi_{T}\right)$ and $G_{Rn}^{⊥,\|}\left(f,\theta_{R},\phi_{R}\right)$ are the gain of the transmitter and receiver antenna, and $\Gamma_{C,On}^{⊥,\|}\left(f,\vartheta_{On}\right)$ is the clutter reflector response, $d_{\mathrm{Ci}n}$, $d_{\mathrm{Cr}n}$ are the distances from transmitter and receiver antennas to the clutter object, respectively. The expression $e^{-j2\pi f\frac{\left(d_{\mathrm{Fi}n}+d_{\mathrm{Fr}n}\right)}{c_{0}}}$ represents the propagation phase for the clutter at the receiver. In general, a metal reflector is known to introduce an additional phase shift of approximately 180 degrees in the reflected wave. However, the exact phase shift caused by a metal reflector can be influenced by factors such as the incident wave’s angle of arrival, polarization, and the specific properties of the metal surface. In the context of clutter and the tag’s reflected signal, our model does not take into account the phase shift generated by themselves. This is because the designed tags in this work are expected to exhibit a similar phase shift to that of the metal reflector due to their grounded structure.

The received signal $Y_{R}\left(f\right)$ in case of clutter is described by (20):

(20) $$Y_{R}\left(f\right)=X_{T}\left(f\right)H_{\mathrm{Clutter}}^{⊥,\|}\left(f\right)$$

The measurement of scattering parameters involves either the reflection coefficient $S_{11}^{⊥,\|}\left(f\right)$ using a single antenna at the reader for both transmission and reception, or the transmission coefficient $S_{21}^{⊥,\|}\left(f\right)$ using two antennas at the reader, where one antenna is dedicated to transmission and the other to reception. In the cluttered scenario, the measurement of scattering parameters from port 1 to port 2 is expressed by Equation (21):

(21) $$\left|S_{\mathrm{Clutter}}^{⊥,\|}\left(f\right)\right|=\left|\frac{Y_{R}\left(f\right)}{X_{T}\left(f\right)}\right|=\left|H_{\mathrm{Clutter}}^{⊥,\|}\left(f\right)\right|$$

## 5. Simulation and Measurement

In this section, our objective is to validate the developed analytical model and assess the performance of both co-polarized and cross-polarized tags. To accomplish this, we conducted simulations using the analytical model and performed measurements for both types of tags in both clutter-free and cluttered environments.

### 5.1. Selection of Reflector Size

**Figure 6:**
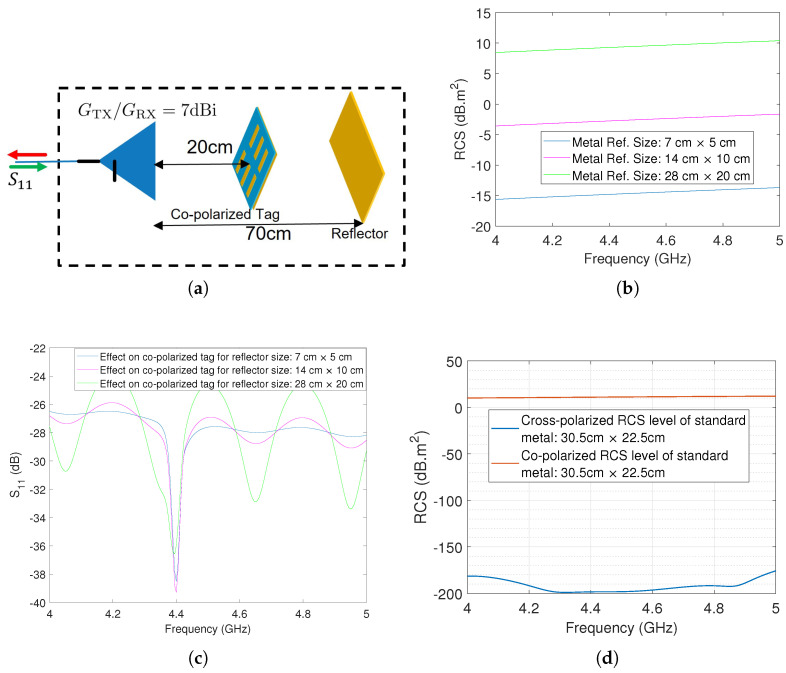
Analysis of worst-case clutter reflector: (**a**) Setup for analytical calculation (**b**) 3 different reflector sizes (**c**) Reflector effect on tag’s response (**d**) Co- and Cross-polarized RCS of available standard reflector in our laboratory.

As a preliminary step to the measurement and simulation phases, we embarked on identifying the most impactful size of a reflector that visibly affects RCS-based tags. To conduct this thorough examination, we opted for a dipole array tag designed to function at 4.4 GHz. To induce multipath effects, we positioned the reflector 70cm away and set the tag at a distance of 20cm, as illustrated in Figure 6a. The subsequent simulation covered a range of reflector sizes, with the primary objective of pinpointing the specific threshold at which the reflector dimensions start to influence the $S_{11}$ response.

The graphs presented in Figure 6b serve as visual representations of the RCS levels across three distinct dimensions. Concurrently, Figure 6c provides insight into the $S_{11}$ response for a reflector positioned 70cm away from the reader antenna. It is worth highlighting that when no reflector is present, a consistent response emerges, devoid of any undulations stemming from constructive and destructive interference. However, with the integration of a reflector measuring 7cm×5cm, noticeable alterations manifest within the $S_{11}$ response attributed to the co-polarized dipole array tag. This dimension represents the minimum size at which a reflector, situated 70cm away, affects co-polarized based tags.

Furthermore, our investigation uncovered that a reflector measuring 28cm×20cm substantially impacts the tag, rendering it undetectable. Given the unavailability of this specific size in our lab, we opted for a metal reflector adhering to the standard PCB dimensions of 30.5cm×22.5cm for further exploration. The simulated co- and cross-polarized RCS levels of the metal reflector are illustrated in Figure 6d.

For the measurement setup, a dual-polarized horn antenna with a gain of 7dBi at 4.5GHz is chosen. This antenna enables the measurement of both co-polarized and cross-polarized tags. Furthermore, the dual-polarized antenna provides sufficient isolation between the cross and vertical polarizations, allowing its usage in a monostatic setup for both types of tags. To serve as the reader, a vector network analyzer (VNA-ZVA 67, R&S, Germany) is utilized. Table 1 provides an overview of the relevant parameters and their corresponding values for the measurement and simulation conducted in this study.

**Figure 7:**
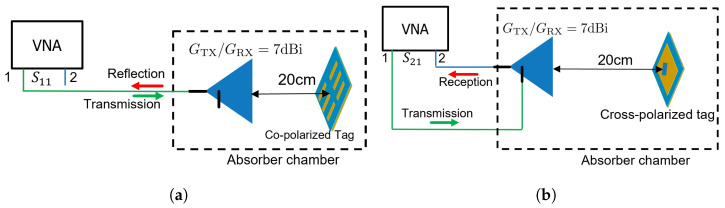
Sketch of the measurement setup and parameters, with “1” representing port-1 and “2” representing port-2: (**a**) $S_{11}$ measurement for co-polarized tags (**b**) $S_{21}$ measurement for cross-polarized tag.

Regarding co-polarized tags, there exist two distinct measurement approaches. The first involves the utilization of two co-polarized antennas. Alternatively, a single antenna with a circulator at its input can be employed to measure $S_{21}$. In contrast, for our measurements, we adopt the approach of using a single antenna to measure $S_{11}$, as depicted in Figure 7a.

In the case of cross-polarized tags, the measurement necessitates the evaluation of $S_{21}$. This requirement arises due to the perpendicular relationship between the polarization of the input signal and that of the output signal. To facilitate this measurement, a dual-polarized antenna or a pair of antennas oriented at a 90° angle to each other can be utilized. Within our laboratory, we possess a dual-polarized antenna, as previously mentioned. Consequently, our measurements involve $S_{11}$ evaluations for co-polarized tags and $S_{21}$ assessments for cross-polarized tags. It is important to note that our focus is not on a direct comparison between $S_{11}$ and $S_{21}$, but rather on examining the detectability. The measurement setup for cross-polarized tags is illustrated in Figure 7b.

### 5.2. Co-Polarized Channel Analytical Simulation and Measurements

In this subsection, measurements were carried out on the co-polarized tags that were previously developed, namely, the dipole array and the square patch. These measurements were conducted in two different conditions: one in a clutter-free environment and the other in the presence of a metal reflector.

The dipole tag exhibits a notch frequency at 4.4 GHz, while the patch tag has a notch at 4.2GHz. It is important to note that both tags are designed to have co-polarization, which means that the incident and reflected signals have the same polarization. Therefore, for the measurement setup, we focused on the vertical polarization only.

**Figure 8:**
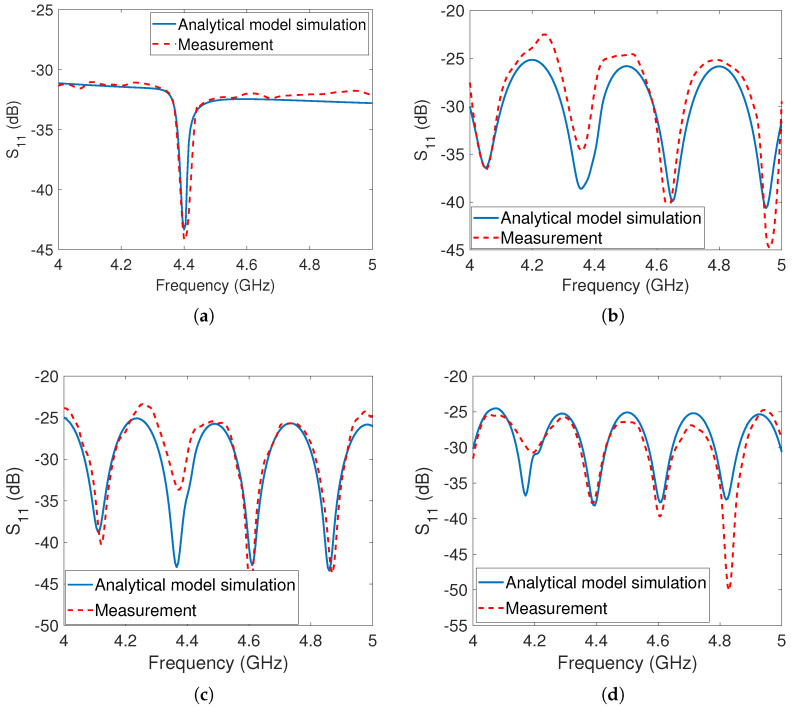
The dipole tag’s analytical simulations and measurements: (**a**) Tag at 20cm from the antenna without metal reflector (**b**) Tag at 20cm and reflector at 70cm from the antenna (**c**) Tag at 20cm and reflector at 80cm from the antenna (**d**) Tag at 20cm and reflector at 90cm from the antenna.

In the initial measurement phase, the dipole array tag was evaluated independently without any obstructing objects present, as shown in Figure 5a. The positioning of the tag was set at a distance of 20cm from the reader. To assess its performance, we performed measurements and simulations using the analytical model, sweeping the frequency range from 4GHz to 5GHz. In particular, we focused on measuring and simulating the $S_{11}$ parameter, which accounts for phase effects and provides insights into the environmental influences on the tag’s response. The results of these measurements and simulations are depicted in Figure 8a, revealing a well-defined notch at 4.4GHz in both the simulated and measured data. Notably, there is a strong agreement between the simulated and measured values, demonstrating the accuracy of the analytical model.

In the second phase of measurement, the reflector was taken into account and positioned behind the tags at various locations, as illustrated in Figure 5b, and the measurement outcomes were recorded. The results of the measurements with the reflector are displayed in Figure 8b–d with the reflector placed at distances of 70cm, 80cm, and 90cm from the reader antenna, respectively. The measurement setups, considering the presence of the metal reflector at all three positions, were simulated using the developed analytical model. It was observed that there was a close match between the simulation and measurement results. However, both the simulation and measurement results revealed the presence of multiple undesirable notches in the $S_{11}$ responses. These unwanted notches caused the original notch at 4.4GHz to disappear, rendering it challenging for the detection algorithm to identify the co-polarized dipole array tag.

The disappearance of the original notch and the appearance of the unwanted notches can be attributed to constructive and destructive interference between the reflected signals from the tag and the reflector. These signals experience different phase delays upon reception by the receiver antenna. Upon observation, it has been noted that an undesired notch is formed as a result of destructive interference when the distance between the tag and the reflector corresponds to an odd integer multiple of ≈$\frac{\lambda }{4}$ at the notch frequency. Conversely, a peak is generated due to constructive interference when the distance between the tag and the reflector corresponds to an even integer multiple of ≈$\frac{\lambda }{4}$ at the peak frequency.

During this phase, our experimental configuration revolved around the strategic placement of a metallic reflector at varying distances, coupled with the evaluation of three distinct transmission angles to scrutinize the impact of the reflector. Throughout these experiments, the co-polarized dipole array tag remained consistently positioned at a distance of 20cm from the reader antenna.

**Figure 9:**
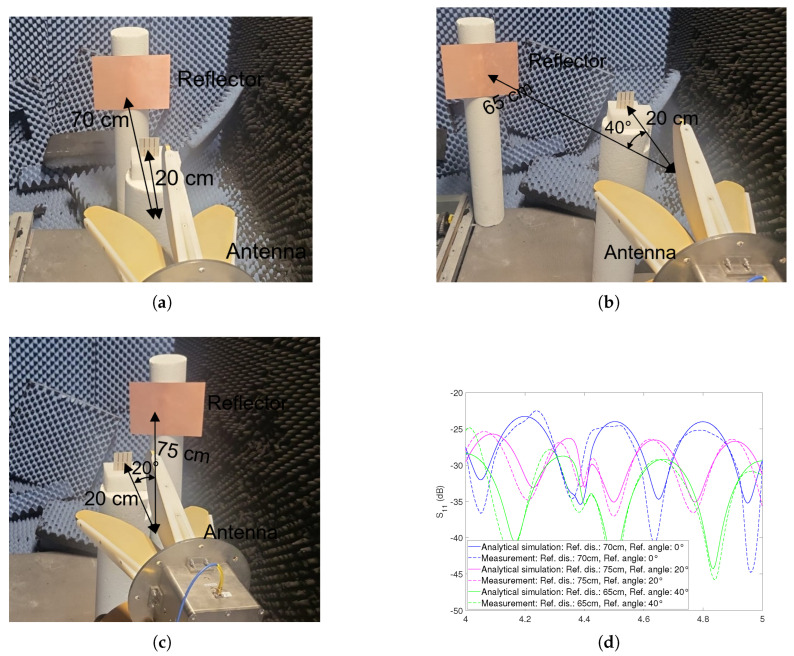
Analytical simulations and measurements of dipole tag: (**a**) Tag at 20cm and reflector at 0°, 70cm away (**b**) Tag at 20cm and reflector at 40°, 65cm away (**c**) Tag at 20cm and reflector at 20°, 75cm away (**d**) $S_{11}$ comparison for reflector at various angles.

The corresponding setups were as follows: The first setup entailed positioning the reflector at 0° and 70cm away, with reference to the reader antenna, as illustrated in Figure 9a. In the second setup, the reflector was placed at 40° and 65cm away, referenced to the reader antenna, as depicted in Figure 9b. Finally, the third setup involved the reflector positioned at 20° and 75cm away, with the reader antenna serving as the reference point, as presented in Figure 9c.

The outcome of these measurements was translated into Figure 9d, and analytical simulations were executed for all three angles. Intriguingly, a notable alignment surfaced between the analytical simulations and the measured $S_{11}$ outcomes, thereby affirming the accuracy of our mathematical model. It is also evident that the tag remains undetectable across all scenarios. Furthermore, the $S_{11}$ level for the reflector positioned at 40° and 65cm is comparatively lower, a phenomenon attributed to the reader antenna’s lower angular gain in that particular setup.

The same method and strategy were used to measure the co-polarized square patch tag in order to analyze the effect of clutter, with the only variation being the angular orientation of the reflector at varying distances. This adjustment was necessary due to the commonality of both tags being co-polarized, thus leading to the anticipation of similar effects.

**Figure 10:**
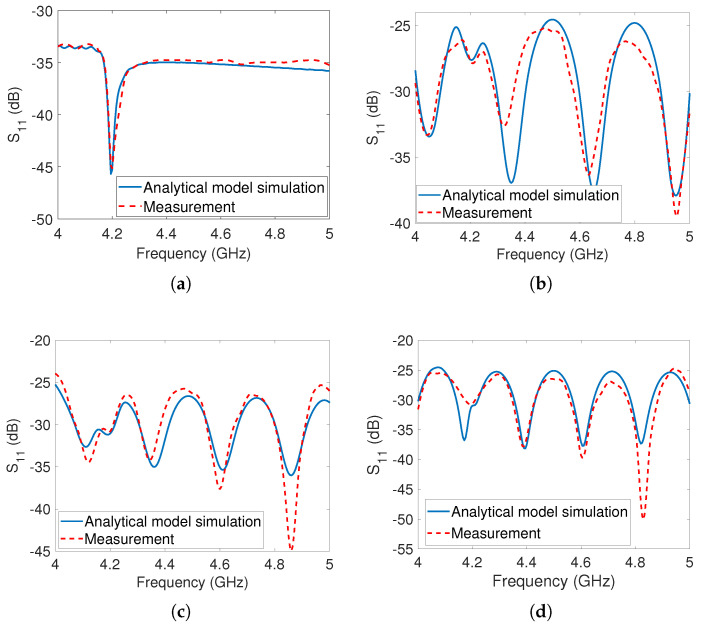
The patch tag’s analytical simulations and measurements: (**a**) Tag at 20cm from the antenna without metal reflector (**b**) Tag at 20cm and reflector at 70cm from the antenna (**c**) Tag at 20cm and reflector at 80cm from the antenna (**d**) Tag at 20cm and reflector at 90cm from the antenna.

In the first measurement, the tag was measured by itself, while in the second measurement, a metal reflector was added to the measurement environment. The square patch tag is single-bit and has a notch at 4.2GHz. The results of the tag measurement without the reflector are displayed in Figure 10a. The measurements of the tag in the presence of the metal reflector are depicted in Figure 10b–d. A clear notch was observed at 4.2GHz when the tag was measured alone, but no real notches appeared in the measurements when the reflector was present, due to the destructive interference between the tag’s reflected signal and the clutter’s reflected signal. The analytical model was used to simulate the tag’s responses in the presence of the metal reflector for different locations, and it was found that they closely matched with the measurement results such as the responses of the dipole array tag. This study of the co-polarized tags shows that detecting a co-polarized tag response in a real environment is extremely difficult.

### 5.3. Cross-Polarized Tag Measurement

Similarly, in this subsection, we focused on the simulation and measurement of the proposed diagonally slotted patch tag, which operates based on co-polarization and cross-polarization. The tag was designed to alter the polarization of the incoming wave, resulting in a notch in the co-polarization plane and a peak in the cross-polarization plane. To measure the reflections of the tag in the cross-polarization plane, we utilized the dual-polarized horn antenna mentioned earlier, which allowed for transmission in one polarization and reception in the orthogonal polarization. The measurement setup, as shown in Figure 5 and Figure 7b, was employed with the exception that both ports of the antenna were utilized.

**Figure 11:**
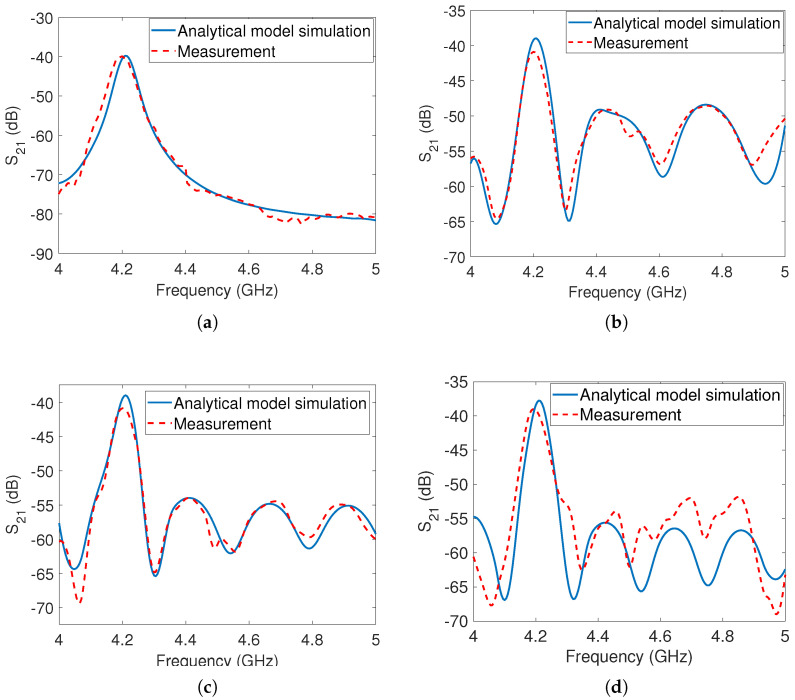
Analytical simulation and corresponding measurements for the cross-polarized tag: (**a**) Tag at 20cm from the antenna without the metal reflector (**b**) Tag at 20cm and reflector at 70cm from the antenna (**c**) Tag at 20cm and reflector at 80cm from the antenna (**d**) Tag at 20cm and reflector at 90cm from the antenna.

In the first measurement scenario, the cross-polarized tag was positioned at a distance of 20cm from the reader antenna. The corresponding analytical simulation and measurement results were presented in Figure 11a, demonstrating a close agreement between the simulation and measurement data.

In the second measurement scenario, the same metal reflector used for the co-polarized tags was placed behind the cross-polarized tag at distances of 70cm, 80cm, and 90cm from the TX antenna, respectively. The analytical simulations and measurement results for these distances are displayed in Figure 11b, Figure 11c, and Figure 11d, respectively. It is evident that the cross-polarized peak remains detectable in all cases, whether or not the metal reflector is present at different distances. This indicates that the metal reflector does not significantly affect the depolarized reflections of the tag, as it is unable to change the polarization of the incident wave. These results confirm the tag’s ability to be detected in the presence of clutter. Furthermore, there is a close match between the simulation and measurement results, validating the accuracy of the analytical model.

## 6. Probability of Detection

In this section, we calculate the probability of detection (PoD) for the measured co-polarized and cross-polarized tags to assess their detectability in a cluttered environment. Notch/peak matched filter detectors are utilized for the detection process, taking into account the signal-to-distortion ratio (SDR) of the measured signal. The SDR considers various factors that can affect the measurement, including noise, calibration mismatch, tag manufacturing defects, and environmental conditions. The algorithm used for PoD calculation has been described and implemented in our previous work [14].

The matched filter algorithm consists of three stages. First, the matched filter response is generated using the RCS response. Next, the measured signal is windowed and convolved with the predefined matched filter response. Finally, a threshold level is selected to make a detection decision.

**Figure 12:**
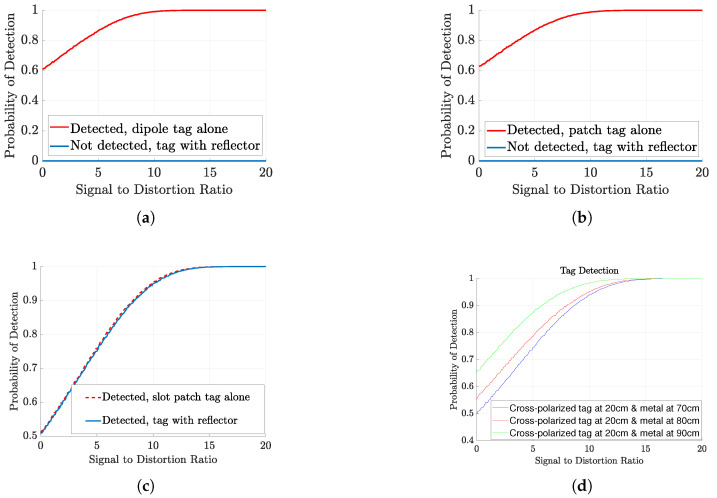
Probability of detection for co- and cross-polarized tags: (**a**) Co-polarized dipole array tag alone at 20cm away from antenna and with reflector placed at 70cm away from the antenna. (**b**) Co-polarized patch tag alone at 20cm away from antenna and with reflector placed at 70cm away from the antenna. (**c**) Cross-polarized patch tag alone at 20cm away from antenna and with reflector placed at 70cm away from the antenna. (**d**) Cross-polarized tag is at 20cm and metal reflector varies over distance.

The detection performance of the co-polarized and cross-polarized tags is evaluated by plotting the probability of detection against the signal-to-distortion ratio. Figure 12a illustrates the PoD of the co-polarized dipole array tag, considering both the presence and absence of the reflector. These results indicate that the co-polarized tag response can only be detected in the absence of the reflector. In the presence of clutter, the PoD performance is significantly degraded due to destructive and constructive interference caused by multipath effects. A similar trend is observed for the square patch tag, as shown in Figure 12b. In contrast, the cross-polarized tag exhibits higher immunity to environmental clutter, as demonstrated by the PoD results shown in Figure 12c. The cross-polarized tag can be successfully detected both in the presence and absence of the metal reflector.

The graphs for the probability of detection in relation to the signal-to-distance ratio (SDR) for various distances are also presented in Figure 12d. It is observed that the probability of detection decreases when the distance is shorter, primarily because the reflector’s higher co-polarized RCS makes it more noticeable. The co-polarized RCS is received by the reader antenna employed in these measurements, which lacks substantial isolation between co- and cross-polarization. As a result, the dynamic range of the peak is decreased, consequently impacting the probability of detection.

## 7. Conclusions

In this paper, we conducted simulations and measurements to examine a major practical limitation of RCS-based co-polarized tags. We found that these tags generate unwanted notches in received signals in cluttered environments that cannot be detected by existing algorithms. To gain a deeper understanding of the clutter effect, we developed a mathematical framework that considers the properties of the reader, tag, and clutter. Simulation results using this framework were compared with measurements for clutter scenarios, and a close agreement was observed, thereby validating the model. In contrast to co-polarized tags, we found that cross-polarized tags were able to detect backscattered signals and were immune to environmental reflections. Using a matched-filter algorithm, we demonstrated that the cross-polarized tag had a high probability of detection even in cluttered environments. However, if there are polarization-alterable objects present in the measurement environment, cross-polarized tags may also show a reduction in detection ability in cluttered environments. Given that a majority of studies concentrate on co-polarized tags, our research establishes the incapability of co-polarized tags to be detected within a cluttered environment. Consequently, researchers can utilize our findings as a point of reference, enabling them to approach the design of chipless tags with a more practical perspective.

## Figures and Tables

**Table 1:** Simulation and measurement parameters.

Parameters	Value
Start frequency	4GHz
Stop frequency	5GHz
Center frequency	4.5GHz
Frequency points	201
IF filter bandwidth	100Hz
Transmit power	−10dBm
Reader antenna gain	$7\;\mathrm{dBi}@0^{\circ }@4.5\;\mathrm{GHz}$, $8\;\mathrm{dBi}@0^{\circ }@4.2\;\mathrm{GHz}$, $6\;\mathrm{dBi}@20^{\circ }@4.2\;\mathrm{GHz}$, $4\;\mathrm{dBi}@40^{\circ }@4.2\;\mathrm{GHz}$
Distance between reader’s TX and tag	20cm
Distance between reader’s TX and metal reflector	70cm, 80cm and 90cm
Reader’s TX and RX isolation	−35dB@4.5GHz

## Data Availability

Data is contained within the article.
